# Population structure of the butternut canker fungus, *Ophiognomonia clavigignenti-juglandacearum*, in North American forests

**DOI:** 10.1002/ece3.332

**Published:** 2012-07-24

**Authors:** K D Broders, A Boraks, A M Sanchez, G J Boland

**Affiliations:** 1 Department of Biological Sciences, University of New Hampshire46 College Rd, Durham, New Hampshire, 03824; 2 School of Environmental Sciences, University of GuelphGuelph, Ontario, Canada, N1G 2W1

**Keywords:** Bayesian clustering analysis, clonality, forest pathogen, invasive species, *Juglans cinerea*, SNPs

## Abstract

The occurrence of multiple introduction events, or sudden emergence from a host jump, of forest pathogens may be an important factor in successful establishment in a novel environment or on a new host; however, few studies have focused on the introduction and emergence of fungal pathogens in forest ecosystems. While *Ophiognomonia clavigignenti-juglandacearum* (*Oc-j*), the butternut canker fungus, has caused range-wide mortality of butternut trees in North America since its first observation in 1967, the history of its emergence and spread across the United States and Canada remains unresolved. Using 17 single nucleotide polymorphic loci, we investigated the genetic population structure of 101 isolates of *Oc-j* from across North America. Clustering analysis revealed that the *Oc-j* population in North America is made up of three differentiated genetic clusters of isolates, and these genetic clusters were found to have a strong clonal structure. These results, in combination with the geographic distribution of the populations, suggest that *Oc-j* was introduced or has emerged in North America on more than one occasion, and these clonal lineages have since proliferated across much of the range of butternut. No evidence of genetic recombination was observed in the linkage analysis, and conservation of the distinct genetic clusters in regions where isolates from two or more genetic clusters are present, would indicate a very minimal or non-existent role of sexual recombination in populations of *Oc-j* in North America.

## Introduction

Invasive species continue to be a major threat to North American forests, and pose an important threat to forest biodiversity and the ecosystem services they provide. Much of the attention related to invasive species has focused on human and animal disease; however, invasive pathogens of plants have the capacity to greatly affect humankind through the devastation of agricultural crops and the destruction of forest ecosystems. It is estimated that invasive plant pathogens cause $21 billion in crop losses each year in the United States, and $2.1 billion of forest products are lost each year to alien pathogens (Pimentel et al. 2005). In addition to economic losses, invasive species pose a significant threat to the health of North American forests and the ecosystem services they provide. Perhaps the most notable fungal invasion into a forest ecosystem is the example of chestnut blight, caused by *Cryphonectria parasistica*, that nearly eliminated all mature American chestnut trees, which was once a dominant hardwood species in the eastern United States. In addition to chestnut blight, several other important exotic fungal diseases have greatly affected the North American forest landscape of the last century. These include, but are not limited to, *Ophiostoma novo-ulmi* causing Dutch elm disease of American elm, *Ophiognomonia clavigigenenti-juglandacearum* causing butternut canker on butternut, *Cronartium ribicola* causing white pine blister rust on white pine, *Phytophthora ramorum* causing sudden oak death on coastal live oak*, Discula destructiva* causing dogwood anthracnose on flowering dogwood, and most recently, *Geosmithia morbida* causing thousand cankers disease, which is damaging black walnuts in the western United States and poses a substantial threat to black walnut plantations throughout North America.

Introduction events or host jump events represent unique evolutionary opportunities for fungal pathogens (Desprez-Loustau et al. 2007), and have the capacity to greatly change the genetic structure of the pathogen's population in comparison with the source population (Stukenbrock and McDonald 2008). For instance, these founder events create an extreme bottleneck that reduces genetic diversity, and new potential hosts provide a strong selection pressure for individuals that can succeed within the context of this new interaction (Sakai et al. 2001; Parker and Gilbert 2004). While the new environments and/or hosts may place selection pressure on the new pathogen, the pathogen also places selection pressure on the host population. In the case of invasive forest pathogens, entire ecosystems can be reshaped by the elimination of a single species, as in the case of chestnut blight (Anagnostakis 1987).

**Figure 1:**
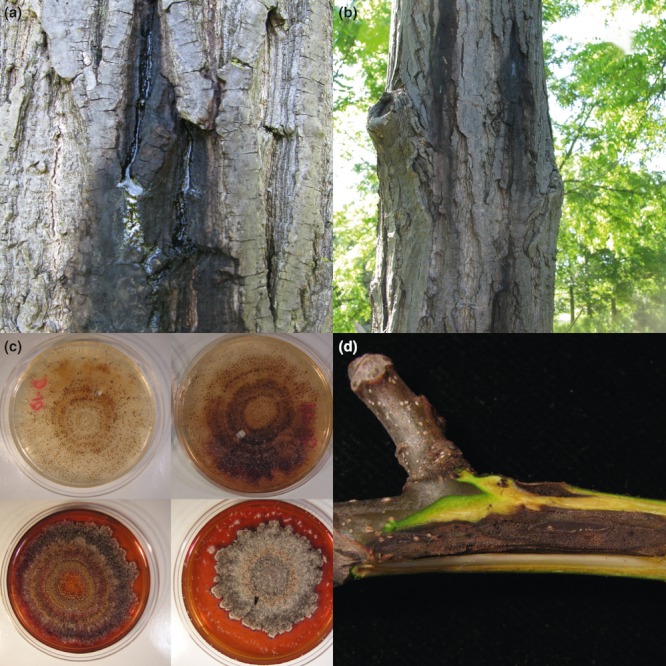
The disease butternut canker on the: (a,b) trunk and (d) stem of butternut trees caused by the (c) fungus *Ophiognomonia clavigignenti juglandacearum*.

In many cases involving invasive forest pathogens, there can be an extended latent period between infection and symptom development, and this delay often results in diseased trees being overlooked. It is therefore quite difficult to determine if the new pathogen was introduced, if subsequent introductions may have occurred, and how far the pathogen has spread since its original introduction. In the case when a source population cannot be identified, it is also possible that the emergent pathogen was derived from an avirulent strain, such as an endopyhyte, on the same host or has made a host jump from a plant species that was either introduced into the new host's range or has migrated into the new host's range due to climate change. A good example of this situation is the case of the butternut canker pathogen, *Ophiognomonia clavigignenti-juglandacearum* (*Oc-J*; Fig. 1), which has caused extensive damage among the butternut population in North America. The first report of butternut canker was in Wisconsin in 1967 (Renlund 1971), and in 1979, the fungus responsible for the disease, *Sirococcus clavigignenti-juglandacearum* (*Sc-j*), was described for the first time (Nair et al. 1979). The sudden emergence of *Oc-j*, its rapid spread in native North American butternuts, the scarcity of resistant trees, and low genetic variability in the fungus (Furnier et al. 1999), point to a recent introduction or emergence of a new pathogenic fungus that is causing a pandemic throughout North America. Recent phylogenetic studies have found that the pathogen that causes butternut canker is actually a member of the genus *Ophiognomonia* and has since been reclassified as *Oc-j* (Broders and Boland 2011). Many of the species in the genus *Ophiognomonia* are endophytes on members of the *Fagales* and more specifically, the *Juglandaceae* or walnut family (Sogonov et al. 2008), which may support the hypothesis of a host jump, where the fungus may have previously been living as an endophyte before coming into contact with butternut. In addition, a recent study from China reported *Sirococcus (Ophiognomonia) clavigignenti-juglandacearum* as an endophyte of *Acer truncatum* (Sun et al. 2011). While the identification was made based on sequence similarity of the ITS region of the rDNA, we have recently gained access to the isolate and found that the isolate is very closely related to *Oc-j* and further phylogenetic and morphological studies will need to be completed to determine if this isolate is in fact *Oc*-*j* or a distinct species. (K. B. Broders, unpubl. data).

It is unclear how long butternut canker disease has existed in North America, but research suggests that *Oc-j* has recently emerged as a pathogen of butternut in North America (Furnier et al. 1999). Since its initial report in 1967, butternut canker was subsequently reported in Canada in Quebec in 1990, in Ontario in 1991 (Davis et al. 1992), and in New Brunswick in 1997 (Harrison et al. 1998) where it was thought to have been present for at least 7 years. The rapid spread of the pathogen into Canada, combined with the devastating effect of this disease, has led to the butternut being designated as an Endangered Species in Canada in 2003 (Neilson et al. 2003). The damaging effect of this pathogen on butternut and the potential danger of an introduction into the commercial walnut growing regions of California demonstrate the importance of understanding as much as possible about the evolutionary history of this pathogen.

At present, there is limited information on genetic variation among isolates of this pathogen from different populations or regions in North America. While a sexual state for the pathogen has not been observed, it does not mean the pathogen is not reproducing sexually at low levels in nature. In fact, many supposedly asexual fungal pathogens show signs of sexual recombination in nature (Taylor et al. 1999). Previous studies failed to detect any variation in RAPD patterns among isolates (Furnier et al. 1999) or polymorphism in five genes including protein-coding genes and rDNA (Broders and Boland 2011), corroborating the prevailing hypothesis that *Oc-j* has recently emerged as a pathogen of butternut and has since reproduced and spread strictly in a clonal state. Unfortunately, these results do not explain observed differences in phenotypic morphology in culture or differences in virulence among isolates observed in previous studies (Ostry and Moore 2008; McKenna et al. 2011). To further investigate the underlying genotypic diversity present among populations of *Oc-j* in North America, Broders et al. (2011b) described a method for the sequencing, assembly, and discovery of single nucleotide polymorphisms (SNPs) from *Oc*-*j*. To identify SNPs, DNA from eight morphologically and geographically distinct isolates was included in the analysis. The results of this project provided 16 SNPs within 5 genomic regions that can be used for further population genetics studies.

The aim of this study was to investigate the genetic diversity of *Oc-j* and the spatial distribution of genotypes in North America. We took advantage of the recently developed SNP markers (Broders et al. 2011b) to perform a genetic analysis of isolates of *Oc-j* from across the range of butternut in North America. We address three questions for the *Oc-j* population sampled in North America: (i) What is the relative importance of outcrossing and clonal reproduction in *Oc-j* populations? (ii) Is there evidence of multiple introduction or emergence events? (iii) Are populations of *Oc-j* geographically (i.e. what is the frequency of short- and long-range dispersal of *Oc-j* genotypes) or genetically structured?

## Material and Methods

### Fungal material

**Figure 2:**
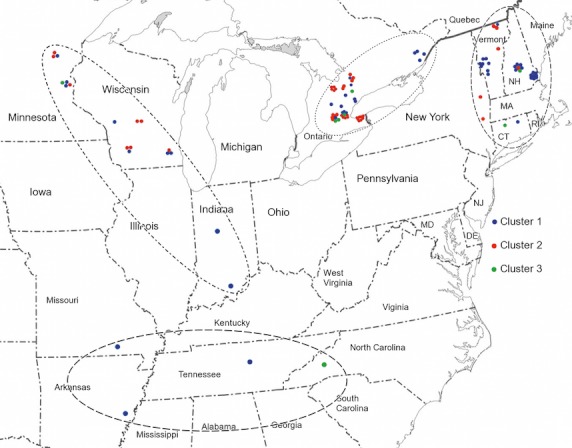
Geographic distribution of *Oc-j* isolates belonging to: (i) one of three genetic clusters (colored dots) determined by structure and PCA analyses, or (ii) one of three distinct geographic populations, denoted by dotted lines (– –, – –, -----) as defined by Hudson's *S*_nn_ test. Each dot represents a single isolate.

Isolates of *Oc-j* were collected from diseased plant tissue from butternut (*Juglans cinerea*), heartnut, (*J. ailantifolia* var. *cordiformis*), and black walnut (*J. nigra)* from locations throughout Ontario in 2009 and 2010 (Table 1). Twenty-seven further isolates of *Oc-j* were collected from Vermont and New Hampshire in 2011. Single spore isolates were established and maintained on PDA agar as previously described (Broders and Boland 2011). The isolates recovered from *J. cinerea* tissues were isolated from infected buds, twig cankers, leaf lesions, and trunk cankers. Isolates from *J. nigra* were recovered from stem and seed pericarp lesions, and isolates recovered from *J. ailantifolia* var. *cordiformis* were recovered from stem cankers and infected buds. In addition, 24 isolates from diverse locations in the United States were provided from Mike Ostry at the Northern Research Station, USDA, St. Paul, MN, and from Amy Rossman at the Systematic Mycology and Microbiology Laboratory, USDA, Beltsville MD. The collection of 101 *Oc-j* isolates represented the following four geographic regions of the U.S. and Canada (Fig. 2); the northeast (NE) region including Connecticut, New Hampshire, New York, and Vermont; Ontario (ONT); the northcentral (NC) region including Indiana, Minnesota, and Wisconsin; and the southeast (SE) region including Arkansas, Missouri, North Carolina, and Tennessee;

**Table 1:** Sampling locations, genetic cluster, geographic region, host, collection date, and haplotype for *Oc-j* isolates collected in North America

Isolate	Cluster^1^	Origin-Location	Region^2^	Host	Collection Date	Haplotype^3^
1375-4a	1	Martell Bnut Block, IN	NC	J. cinerea	8/12/08	H1
1378-3	1	Hoosier NF, IN	NC	J. cinerea	8/14/08	H12
1339-6	1	Hay Creek, MN	NC	J. cinerea	4/3/01	H2
AR 4532	1	Hay Creek, MN	NC	J. cinerea	4/3/01	H2
AR 4536	1	Lakewood Rd, WI	NC	J. cinerea	6/5/03	H2
ATCC 36624	1	Wisconsin (type culture)	NC	J. cinerea	1979	H2
1343-408-1	1	White Water, WI	NC	J. cinerea	2/13/02	H5
1343-408-3	1	White Water, WI	NC	J. cinerea	2/13/02	H5
MER22	1	Meredith, NH	NE	J. cinerea	4/21/11	H1
MER25	1	Meredith, NH	NE	J. cinerea	4/22/11	H1
MER27	1	Meredith, NH	NE	J. cinerea	4/22/11	H1
MER5	1	Meredith, NH	NE	J. cinerea	4/22/11	H1
JRF1271	1	Jericho, VT	NE	J. cinerea	4/22/11	H11
JRF1272	1	Jericho, VT	NE	J. cinerea	4/22/11	H11
UN22B1	1	Durham, NH	NE	J. cinerea	4/24/11	H13
UN22B2	1	Durham, NH	NE	J. cinerea	4/24/11	H13
UNH21	1	Durham, NH	NE	J. cinerea	4/24/11	H13
UNH22	1	Durham, NH	NE	J. cinerea	4/24/11	H13
UNH23	1	Durham, NH	NE	J. cinerea	4/24/11	H15
SNAKE	1	Charlotte, VT	NE	J. cinerea	2/17/11	H33
CHAR2	1	Charlotte, VT	NE	J. cinerea	2/22/11	H36
WILL2	1	Williston, VT	NE	J. cinerea	2/22/11	H36
MONK1	1	Monkton, VT	NE	J. cinerea	2/17/11	H38
MER21	1	Meredith, NH	NE	J. cinerea	4/22/11	H40
UN21B3	1	Durham, NH	NE	J. cinerea	4/24/11	H5
AR 4541	1	Guilford, CT	NE	J. cinerea	2001	H5
FOX41	1	Charlotte, VT	NE	J. cinerea	2/27/11	H6
NEWHAV	1	New Haven, VT	NE	J. cinerea	2/17/11	H6
FOX42	1	Charlotte, VT	NE	J. cinerea	2/27/11	H7
WOOD1	1	Durham, NH	NE	J. cinerea	4/24/11	H8
WOOD2	1	Durham, NH	NE	J. cinerea	4/24/11	H8
WOOD21	1	Durham, NH	NE	J. cinerea	4/24/11	H9
HN1	1	Guelph, ON	ONT	J. ailantifolia	4/23/09	H1
74-6	1	Barrrie, ON	ONT	J. cinerea	7/12/12	H1
74-7	1	Barrrie, ON	ONT	J. cinerea	7/13/12	H1
76-2	1	Barrrie, ON	ONT	J. cinerea	7/15/12	H1
76-3	1	Barrrie, ON	ONT	J. cinerea	7/16/12	H1
76-4	1	Barrrie, ON	ONT	J. cinerea	7/17/12	H1
259L-LB11	1	Guelph, ON	ONT	J. cinerea	4/25/09	H1
B6L-TB12	1	Guelph, ON	ONT	J. cinerea	4/25/09	H1
P-013	1	Guelph Lake, ON	ONT	J. cinerea	8/8/08	H10
P-046	1	Big Rideau Lake, ON	ONT	J. cinerea	9/17/08	H14
P-010	1	Simco Lake, ON	ONT	J. cinerea	8/3/08	H2
SCJ1	1	York, ON	ONT	J. cinerea	3/15/11	H23
P-045	1	Big Rideau Lake, ON	ONT	J. cinerea	9/16/08	H26
P-029	1	Simco Lake, ON	ONT	J. cinerea	8/14/08	H33
P-043	1	Charleston Lake, ON	ONT	J. cinerea	9/11/08	H34
P-037	1	Brockville, ON	ONT	J. cinerea	8/28/08	H37
P-019	1	Hockley Valley PNR, ON	ONT	J. cinerea	8/22/08	H37
P-017	1	Conestoga Lake, ON	ONT	J. cinerea	8/14/08	H38
SCJ3	1	York, ON	ONT	J. cinerea	3/15/11	H4
P-005	1	Simco Lake, ON	ONT	J. cinerea	7/18/08	H41
WB3-16	1	Cambridge, ON	ONT	J. cinerea	5/5/09	H42
BW1-1	1	Cambridge, ON	ONT	J. nigra	10/5/09	H1
AR 4539	1	Smithville, TN	SE	J. cinerea	4/14/01	H39
AR 4538	1	St. Francis NF, AK	SE	J. cinerea	4/11/01	H39
1368-1c	1	Oregon Co., MO	SE	J. cinerea	3/16/07	H43
1339-13	2	Hay Creek, MN	NC	J. cinerea	4/3/01	H16
AR 4535	2	Lakewood Rd, WI	NC	J. cinerea	6/5/02	H16
1362-5	2	Rum River SF, MN	NC	J. cinerea	4/13/06	H16
1363-7b	2	Rum River SF, MN	NC	J. cinerea	4/13/06	H16
AR 4533	2	White Water, WI	NC	J. cinerea	2/13/02	H16
AR 4534	2	Lakewood Rd, WI	NC	J. cinerea	6/5/02	H24
1382-1a	2	Mauston, WI	NC	J. cinerea	8/22/08	H24
1382-1b	2	Mauston, WI	NC	J. cinerea	8/22/08	H24
1235-4	2	Essex Co., NY	NE	J. cinerea	6/30/93	H16
1210-10	2	Ulster Co., New York	NE	J. cinerea	6/30/93	H16
ORANG1	2	Orange, VT	NE	J. cinerea	2/17/11	H17
MER32	2	Meredith, NH	NE	J. cinerea	4/22/11	H22
DL101	2	Derby Line, VT	NE	J. cinerea	2/24/11	H35
DL81	2	Derby Line, VT	NE	J. cinerea	2/24/11	H45
WB3-1	2	Cambridge, ON	ONT	J. cinerea	5/5/09	H18
76-1	2	Barrrie, ON	ONT	J. cinerea	7/14/12	H19
WB1-4	2	Cambridge, ON	ONT	J. cinerea	5/5/09	H20
Bud2-3	2	Guelph, ON	ONT	J. cinerea	4/14/09	H20
GA5-1	2	Guelph, ON	ONT	J. cinerea	4/14/09	H20
S1L-LB12	2	Guelph, ON	ONT	J. cinerea	4/25/09	H20
P-034	2	Bradford, ON	ONT	J. cinerea	8/26/08	H21
GA 1-1	2	Guelph, ON	ONT	J. cinerea	4/14/09	H24
P-008	2	Simco Lake, ON	ONT	J. cinerea	7/30/08	H3
76-11	2	Barrrie, ON	ONT	J. cinerea	7/19/12	H44
76-5	2	Barrrie, ON	ONT	J. cinerea	7/18/12	H44
958L-TB11	2	Guelph, ON	ONT	J. cinerea	4/25/09	H44
P-003	2	Simco Lake, ON	ONT	J. cinerea	7/17/08	H44
72-1-5	2	Niagara-by-the-lake, ON	ONT	J. cinerea	6/8/10	H25
72-3-1	2	Niagara-by-the-lake, ON	ONT	J. cinerea	6/7/10	H25
72-4-2	2	Niagara-by-the-lake, ON	ONT	J. cinerea	6/7/10	H25
72-7-2	2	Niagara-by-the-lake, ON	ONT	J. cinerea	6/7/10	H25
72-1-1	2	Niagara-by-the-lake, ON	ONT	J. cinerea	6/7/10	H44
72-1-3	2	Niagara-by-the-lake, ON	ONT	J. cinerea	6/7/10	H44
72-1-4	2	Niagara-by-the-lake, ON	ONT	J. cinerea	6/7/10	H44
BW-2	2	Cambridge, ON	ONT	J. nigra	10/5/09	H16
BW2-1	2	Cambridge, ON	ONT	J. nigra	10/5/09	H16
1365-4	3	Rum River SF, MN	NC	J. cinerea	4/13/06	H28
MER31	3	Meredith, NH	NE	J. cinerea	4/22/11	H27
AR 4540	3	Chester, CT	NE	J. cinerea	2001	H29
DL102	3	Derby Line, VT	NE	J. cinerea	2/24/11	H31
70-BW1	3	Guelph, ON	ONT	J. nigra	6/15/09	H27
AR 4537	3	Asheville, NC	SE	J. cinerea	4/24/01	H30
WB-22	-	Cambridge, ON	ONT	J. cinerea	7/8/09	H32

1 Genetic cluster assignment was determined by Bayesian analysis in the program Structure and PCA analysis in the program adegenet.

2 Isolates were collected from four geographic regions; NC (Northcentral) Indiana + Minnesota + Wisconsin: NE (Northeast), Connecticut + New Hampshire + New York + Vermont: ONT (Ontario): and SE (Southeast), Arkansas + Missouri + North Carolina + Tennessee.

3 Isolates that were identical at all 17 SNPS were considered the same haplotype.

### DNA extraction and amplification

Isolates of *Oc-j* were grown on cellophane-covered PDA for 7–10 days, mycelia were collected and DNA was extracted using the MoBio Power Plant DNA extraction kit (Mo Bio Laboratories Inc., Carlsbad, CA). DNA purity and quantity were estimated using a Nanodrop photospectrometer.

Seventeen SNPs located within five genomic regions including the 16 SNPs previously developed by Broders and Boland (2011) and an additional SNP identified during the course of this study were used to genotype each isolate. The SNP polymerase chain reactions (PCR) were performed in a 50-μL reaction consisting of 10 μL of 5× Green GoTaq reaction buffer (Promega Corp., Madison, WI), 5 μL of 25 mmol/L MgCl_2_, 1 μL containing 10 mmol/L each dNTP, 0.25 μL of GoTaq *Taq* polymerase, 5 μL each of 5-μmol/L concentration of forward and reverse primers, 2 μL of DNA at a concentration of 10 ng/μL, and 21.75 μL of sterile deionized water. PCR parameters were 95°C for 5 min; followed by 35 cycles of 95°C for 1 min, 54°C for 1 min, 72°C for 1 min; and completed with 72°C for 5 min followed by 4°C. PCR products were purified using Qiaquick spin columns (Qiagen Inc., Valencia, CA). For sequencing, 2 μL of 5 pmoles/μL primer was added to 2 μL of purified PCR product (20 ng/μL). Amplified products were sequenced with the BigDye version 3.1 ready reaction kit (Applied Biosystems, Foster City, CA) on an ABI 3730 automated sequencer at the University of Guelph Genomics Facility or the Hubbard Center for Genome Studies at the University of New Hampshire. Sequencing chromatograms were visualized, and sequences were aligned and edited using BioEdit (Hall 1999).

### Data analysis

The sequences of the five genomic regions in which SNPs were identified were aligned using ClustalW (Thompson et al. 1994) and then concatenated and collapsed into unique haplotypes using the SNAP Combine and SNAP Map functions, respectively, in SNAP workbench 2.0 (Price and Carbone 2005). The likelihood of copies of haplotypes resulting from sexual reproduction, by calculating the probability *P*gen(f), taking into account departure from Hardy–Weinberg equilibrium, was estimated for the sample set using GenClone 1.0 (Arnaud-Haond et al. 2007). Tests for neutrality were completed to determine whether observed genetic variation was consistent with the hypothesis that the majority of polymorphisms contributing to genetic variability are selectively neutral (Kimura 1983). Tajima's *D*; Fu and Li's *D* and *F*; and Fu's *F* neutrality statistics were tested using DnaSP version 5 (Rozas et al. 2003). Tests for linkage disequilibrium (LD) were assessed using MultiLocus 1.2 (Agapow and Burt 2001) and used to detect the nonrandom association of alleles at different loci (Slatkin 1994). LD was assessed with each haplotype within a genome fragment considered an allele at that locus. Significance was assessed using 1000 randomizations of the data set to create a null hypothesis. Genotypic diversity for each region was estimated by calculating the haplotype diversity (Hd) using Dna SP version 5 (Rozas et al. 2003).

Phylogenetic analysis based on haplotypes was performed using the neighbor joining (NJ) method (Kimura two-parameter distance calculation) in MEGA version 4.0. All positions were included in the analysis and relative support for the branches was estimated with 1000 bootstrap replications. In addition, a set of 95% plausible haplotype networks, for the *Oc-j* population in North America, connecting the haplotypes by mutational steps, was constructed using statistical parsimony in the program TCS (Clement et al. 2000).

As the origin of *Oc-j* is not well understood, it is possible that the fungus emerged from an alternate host and/or was introduced on more than one occasion. Therefore, the genetic structure of the population may not coincide with geographic proximity of individuals, but rather are clustered based on their genetic relatedness. To test the geographic and genetic structure of populations, we used three methods. The first was a series of nonparametric tests applied in a hierarchical manner by the program SNAP Map (Aylor et al. 2006) and two programs performing Hudson's test *S*_nn_ (Hudson and Kaplan 1985) and Permtest (Hudson et al. 1992), using 1000 permutations to determine a null distribution to assess significance of results. First, sequences from six geographically distinct populations in North America were tested in pairwise comparisons. Each pair of two locations with no significant genetic difference between them was collapsed into a single population for subsequent tests, and then another round of pairwise comparisons was completed. This continued until only locations that were significantly different, and geographically structured populations, remained.

We tested for the existence of divergent genetic pools of *Oc-j* in North America using a Bayesian analysis using the program Structure 2.2 (Pritchard et al. 2000) and a genetic multivariate analysis in adegenet (Jombart 2008) to detect genetically differentiated groups corresponding to independent introductions. These methods avoid the clustering of individuals on a priori information, such as geographic locations, that may mix divergent genetic lineages introduced in the same area and may hinder the detection of admixture events among these lineages.

The analysis with Structure can be used to estimate parameters independently of the posterior probability distribution of allele frequencies. Parameters are estimated under the null model of panmixia, where each locus is at Hardy-Weinberg equilibrium and independent of the others. Using the admixture model, we estimated the number of genetic clusters, between *K =* 1 and *K* = 6, to which each haplotype should be assigned. Ten independent runs were conducted to evaluate the consistency of the results across runs. All runs had a burn-in period of 100,000 iterations with a run length of 100,000 iterations following the burn-in. The method developed by Evanno et al. (2005) was used to determine the optimum number of populations (*K*) empirically determined by comparing posterior distribution likelihoods among runs. Although Structure has been used to study genetic relationships among races, cryptic species or to detect regional substructures in fungal pathogen species, violation of hypotheses such as linkage disequilibrium in clonal subgroups can sometimes lead to incorrect assignments. We therefore, also used a principal component analysis (PCA) to investigate the genetic structure of the *Oc-j* population in North America. As PCA is independent of any genetic hypotheses, such as Hardy–Weinberg equilibrium, it is suitable for the analysis of clonal or partially clonal species. PCA analysis was performed using the Adegenet package under the R-software. For both the Bayesian and PCA analyses, only the clone-corrected data set was used to give identical weight to each multilocus genotype.

Clonal diversity within each cluster was evaluated as the clonal richness (*R*), the Simpson evenness index (*V*), and the complement of the slope of the Pareto distribution of clonal membership in the program GenClone. These measures are recommended as the most parsimonious set of non-redundant indices of clonal diversity (Arnaud-Haond et al. 2007). In addition, analysis of molecular variance (AMOVA) was conducted on the resulting genetic and geographic clusters using Arlequin (Excoffier et al. 2005). Analysis of molecular variance is a method of partitioning genetic diversity into within-population and among-population components for detecting population differences. Two groups of “populations” were analyzed. The first group of populations consisted of the three genetic clusters that were the result of the Bayesian and PCA analyses. The second group consisted of the three significantly different geographic populations detected using Hudson's test *S*_nn_ (Hudson 2000) and Permtest in the program SNAP Map.

## Results

### Haplotype analysis

Based on the combination of 17 polymorphic loci within non-coding regions of the *Oc-j* genome, 45 haplotypes were identified among 101 isolates analyzed. Eighteen (40%) of the haplotypes were found to have multiple isolates with three haplotypes (H1, H16, and H44) having eight or more isolates making up 32% of the isolates sampled. Haplotype H1 had the most isolates (15) and included isolates from the NE, NC, and ONT populations. H16 had the second largest number of isolates (9) and also included isolates from the NE, NC, and ONT populations. In contrast, all eight isolates of H44 were recovered from Ontario. All neutrality tests were non-significant, indicating these polymorphic loci are selectively neutral. Tests for linkage disequilibrium among the five regions found significant LD overall (index of association [*I*_A_] = 0.59, *P* = 0.011), and in pairwise tests of the five genomic regions, significant LD was also detected ([*I*_A_] = 0.62 *P* = 0.023).

### Population structure analysis

The initial test with Hudson's *S*_nn_ compared isolates from the six populations in the U.S. and Canada. There was no significant difference between the populations from the northeast and southeast (*P* = 0.12), Wisconsin, Minnesota, and Indiana (*P* = 0.34), and Ontario remained its own group as there was no significant difference (*P* = 0.23) between the south-central and eastern Ontario populations (Fig. 2). A second *S*_nn_ test was then performed on the three larger groups created from the pooling of groups that were not significantly different in the first run. In this test, all pairwise comparisons of the three groups were significantly different (*P* < 0.0001). The result was a putative North American population of three groups designated northeast/southeast (NE/SE), northcentral (NC), and Ontario (ON) (Fig. 2).

**Figure 3:**
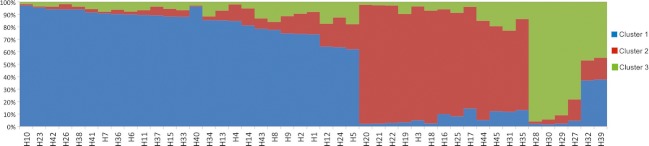
Structure bar plot indicating *q*-value as probability (*y*-axis) of each haplotype belonging to one of three groups. Each vertical bar represents one haplotype.

When the clone-corrected dataset was analyzed in Structure, the posterior probabilities of the allele frequencies among clusters were best explained with a grouping into three clusters. Assuming a quality threshold of *q>* 0.75 for assignment to a particular cluster, 84% of the isolates belonged to only one cluster, indicating that the three clusters were highly differentiated (Fig. 3). Twenty-six haplotypes, including two of the most frequent haplotypes H1 and H2, were grouped into cluster 1 consisting of isolates from the NE, ONT, NC, and SE populations (Figs. 2, 3). Cluster 2 was composed of 13 haplotypes, including the second and third most frequent haplotypes H16 and H44, located mainly in Ontario and the Northeastern U.S. (Figs. 2, 3). Cluster 3 consisted of four haplotypes, and while this cluster is much smaller than the first two, isolates were recovered from a similar geographic range including isolates from the NE, ONT, NC, and SC populations (Figs. 2, 3).

**Figure 4:**
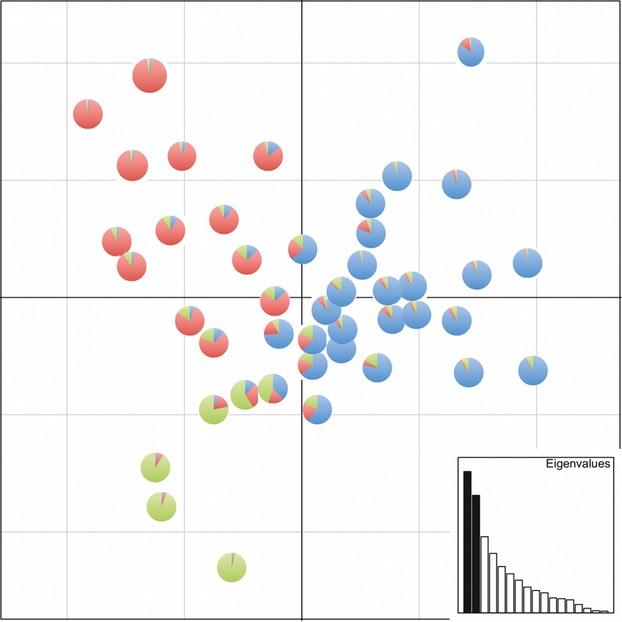
Coordinates for the 45 haplotypes of *Oc-j* sampled from North America on the two main axes of the PCA. Pie charts give the probability of assignment for each genotype to the three genetic clusters tested by Structure. Genetic clusters C1, C2, and C3 are represented by blue, red, and green, respectively.

The PCA analysis was able to discriminate three distinct groups similar to the Structure results. Two groups of haplotypes were separated on the first axis (Fig. 4). One group with positive coordinates on the first axis (PC1) was composed of the genotypes that corresponded to cluster 1 defined by Structure, and the group on the negative side of the first axis was composed of haplotypes that corresponded to clusters 2 and 3 as defined by Structure (Fig. 4). Cluster 2 and 3 were further separated along axis 2. As the Structure clustering output is supported by the results of the multivariate analysis, this indicates that the assignment obtained by Structure is reliable despite the deviations from the assumptions of the model. Therefore, further analyses were conducted by grouping isolates and/or haplotypes into three clusters obtained using PCA and Structure.

**Figure 5:**
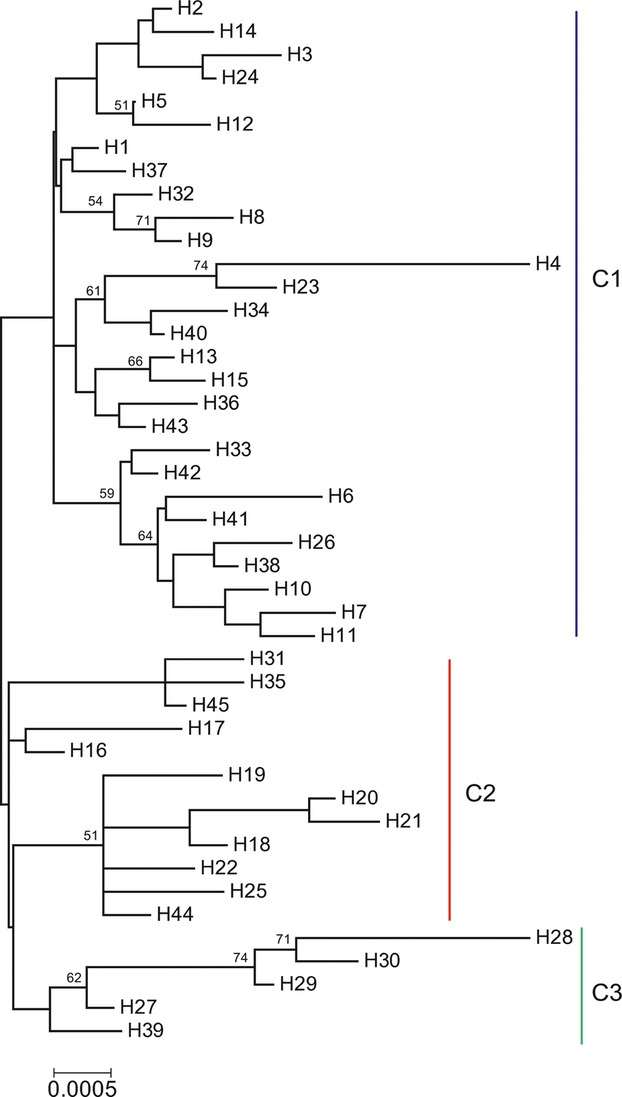
The unrooted tree from the neighbor-joining analysis of five genomic regions containing 17 SNPs from the 45 haplotypes of *Oc-j*. Bootstrap replicate values greater than 50% are shown above the branch. The three major clades correspond to the three genetic clusters (C1, C2, and C3) determined in Structure and PCA analyses.

**Figure 6:**
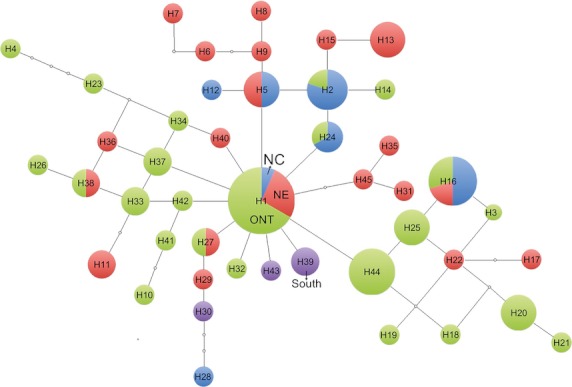
Most parsimonious haplotype network for 45 haplotypes of *Oc-j* recovered from across North America, derived from 17 SNPs. Each of the four larger regions is shown for each haplotype: NC (Blue), Indiana + Minnesota + Wisconsin: NE (Red), Connecticut + New Hampshire + New York + Vermont: ONT (Green), Ontario: and South (Purple), Arkansas + Missouri + North Carolina + Tennessee. Sizes of haplotype circles indicate frequencies of the haplotypes. Each line represents a single nucleotide mutation, and small empty circles represent unsampled haplotypes.

The phylogenetic tree developed from the neighbor-joining analysis of the combined alignment of the five genomic regions from the 45 haplotypes found a similar clustering pattern to that of the Structure and PCA analyses (Fig. 5). However, bootstrap values at the major nodes were weak, and some homoplasy appears to be present in the haplotype map (Fig. 6). The haplotype map grouped haplotypes into groups similar to the neighbor-joining analysis. The haplotype map also provided some evidence of geographic expansion of several haplotypes that were found in more than one region. The haplotype diversity (Hd) for the Northeast, Northcentral, and Southeast region and Ontario were 0.96, 0.88, 0.83, and 0.92, respectively. This would indicate a high level of haplotype diversity across all regions, with the highest observed diversity in the Northeast region, as Hd approaches 1 as diversity increases.

Both the three-population geographic structure observed with the *S*_nn_ test and the three-population genetic structure found with the Bayesian and PCA analyses were tested with AMOVA using Arlequin. The best population structure as determined by *F*_ST,_ smallest within population variation and largest among population variation, was observed for the three-population model of admixed individuals from different regions (Table 2). However, the three-region model had a significant *F*_ST_ = 0.12 and all pairwise *F*_ST_ values were significant (Table 3).

**Table 2:** Analysis of molecular variance for *Oc-j* populations determined by Bayesian and PCA analyses to determine genetic structuring and Hudson's *S*_nn_ test statistic for geographic subdivision

	Genetic clusters		*S*_nn_ clusters	
		
Source of variation	df	Total variance (%)	df	Total variance (%)
Among populations	2	31.72	2	12.49
Within population	98	68.28	98	87.51
Fixation index (*F*_ST_)		0.41		0.12

**Table 3:** Pairwise *F*_ST_ values calculated for both the three-population geographical model observed with the *S*_nn_ test in SNAP workbench and the three-population genetic structure model observed using the PCA analysis in adegenet and Bayesian analysis in structure. Significant (*P* < 0.05) *F*_ST_ values are denoted by (*)

	Geographic population		
	
Genetic cluster	1	2	3
1		0.09*	0.16*
2	0.46*		0.16*
3	0.32*	0.36*	

### Genetic properties of the three genetic clusters

All genetic clusters had a clonal structure, as shown by the number of repeated haplotypes, or haplotypes that were only distant by 1 allele (Table 4). The Simpson evenness index was similar for the two largest clusters, as both were near 0.83. Only clusters 1 and 2 contained enough isolates to calculate the slope of the Pareto distribution. Isolates in both clusters 1 and 2 had a slightly skewed distribution (β = 1.52 and 1.53), where most of the haplotypes showed comparable frequencies with the exception of one or two more frequent haplotypes. This was likely a result of the fact that the two largest clones H1 and H16 were members of genetic clusters 1 and 2, respectively, and the remaining haplotypes consisted of only 1 or 2 isolates. The *r*_d_ tests on the complete dataset and clone-corrected dataset rejected the null hypothesis of recombination (*P* < 0.001). Taken together, these analyses point to a strongly clonal reproduction regime for each of the genetically distinct clusters, using both the complete and clone-corrected datasets.

**Table 4:** Genetic features of the entire population of *Oc-j* and three clusters of *Oc-j* isolates determined by the PCA and Bayesian clustering analyses. These non-redundant indices of clonal diversity were calculated using the program GenClone

Statistics	Whole population	Cluster 1	Cluster 2	Cluster 3
Isolates	101	57	36	6
No. distinct haplotypes	45	27	13	5
Haplotypes different by 1 allele		5	9	2
Clonal richness	0.44	0.46	0.34	0.8
Simpson evenness (*V*)	0.91	0.82	0.83	0.95
c(pareto)	1.7	1.57	1.56	NA
Slatkin exact test *P*-value		0.76	0.18	0.22

### Spatial distribution of genetic clusters

**Figure 7:**
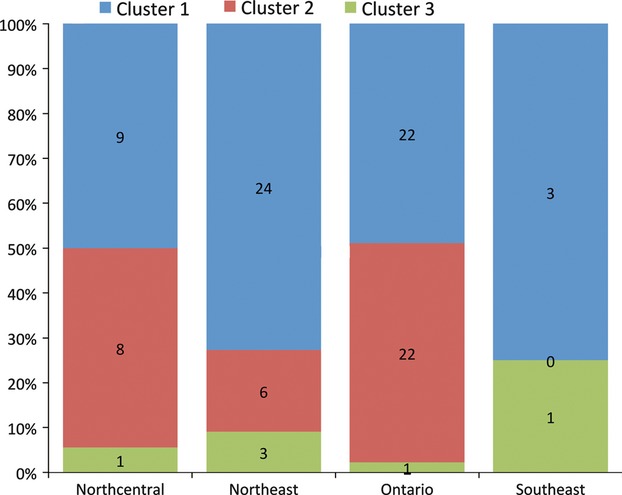
Frequency of isolates from each of the three genetic clusters, determined by assignment tests in the program Structure and PCA analysis, for each region in North America where *Oc-j* was collected. Numbers in each segment represent the number of isolates in each cluster in each region.

The frequency of isolates from each of the genetic clusters among regions was calculated using all isolates. Isolates belonging to cluster 1 were found in all four regions of North America and were the most abundant genetic cluster in each region (Fig. 7). Isolates belonging to cluster 2 were found in all regions except the southern region and represented greater than 44% of the individuals in Ontario and the Northcentral region versus only 18% of individual in the Northeast. While composing only a small proportion of individual in each region, cluster 3 was present across North America.

## Discussion

This use of SNP markers provides the first comprehensive description of the genetic diversity of the North American population of *Oc-j*. While numerous reports and papers have been published on the progression of the butternut canker epidemic since its first appearance in the 1960s (Orchard 1984; Davis et al. 1992; Innes and Rainville 1996; Ostry 1997; Ostry et al. 1997; Harrison et al. 1998; Ostry and Woeste 2004; Ostry and Moore 2007), only two other studies have attempted to evaluate the genetic diversity of the pathogen (Furnier et al. 1999; Broders and Boland 2011). Previous genetic studies of *Oc-j* suffered from a lack of variability in the markers being analyzed. This was likely due to the highly conserved nature of the genome of a recently introduced clonal fungus and the lack of resolving power of the markers chosen in previous studies. Using RAPD DNA fingerprints obtained with dominant markers, Furnier et al. (1999) were not able to detect any differences among isolates of *Oc-j* from several locations in the United States. Another study by Broders and Boland (2011) targeted five genes and found no diversity among the ITS, β-tubulin, actin, calmodulin, and EF1-α from 24 isolates of *Oc-j* from across North America. In contrast to these studies, we found a much larger than expected rate of genetic variability among the entire population from North America with 45 distinct haplotypes identified among the 101 individuals sampled, including individuals used in the previous study by Broders and Boland (2011). These results demonstrate the advantage of using neutral SNPs found in non-coding portions of the genome versus dominate markers or genes that may not accumulate mutations at the same rate as non-coding regions, when working with recently emergent clonal organisms. This study also helped to provide strong evidence for the clonal nature of this organism as hypothesized in previous studies (Furnier et al. 1999; Broders and Boland 2011). In addition, we were able to identify three distinct clonal lineages within the North American population of *Oc-j* using both Baysian and PCA analyses of the SNP data.

### Population structure of Oc-j in North America

The low level of nucleotide variation and the high proportion of low-frequency haplotypes detected in the *Oc-j* populations in North America support the inference of a recent introduction and subsequent haplotype expansion. However, the genetic structure deduced from the SNP genotyping was not consistent with the hypothesis that a single invading clone of *Oc-j* colonized the butternut populations of North America. Instead, our results indicate that the emergence of *Oc-j* resulted from the admixture of three genetically differentiated groups of isolates (Figs. 5). This population structure raises an important question about the emergence dynamics of the *Oc-j* populations. Do the three genetically differentiated clusters represent distinct introductions or emergence events? Based on the results from the phylogenetic analyses (Figs. 5, 6), Bayesian analysis in Structure and PCA (Figs. 3, 4) and subsequent identification that these three genetically distinct groups had a clonal structure, we can deduce that there were likely at least three independent introduction or emergence events. However, while the populations are genetically structured, there is also a significant subdivision among geographic regions. This would indicate that the different clonal lineages may have emerged at different locations and/or times, or specific clonal lineages are more adept at long distance transmission, either via insect vector (Katovich and Ostry 1998; Halik and Bergdahl 2002; Stewart et al. 2004) or infected seed (Innes and Rainville 1996; Broders and Boland 2010). In addition, the origin of *Oc-j* is unknown, as the isolate has not been identified outside North America. Therefore, it is possible that *Oc-j* is resident to North America as an endophyte or minor pathogen of a different forest species and made a host jump when it came in close proximity to butternut, as has been observed with several important crop pathogens that originated from wild plant species (Couch et al. 2005; Zaffarano et al. 2008). The other potential scenario is that *Oc-j* was introduced on a foreign plant species, such as the closely related Japanese walnut (*Juglans ailantifolia*) commonly known as heartnut, which is a close relative of butternut and has been known to hybridize with butternut. While *Oc-j* does not kill Japanese walnut, it has been isolated from small branch cankers on the tree. The one isolate from *J. ailantifolia* in this study was found to belong to haplotype H1, the most frequently recovered haplotype, which also appears to be ancestral to many of the other haplotypes. Further isolation and genotyping of *Oc-j* from *J. ailantifolia* will help determine the role of this tree in the introduction and spread of *Oc-j*.

A third potential scenario is the introduction of the fungus on wood or lumber products imported into the United States. Major ports of entry for wood products into the eastern North America include several locations along the east coast as well as through the St. Lawrence Seaway, which officially opened in 1959, marking the first arrival of an oceangoing ship in the Duluth-Superior harbor in Minnesota. While the St. Lawrence Seaway has been credited with the introduction of many aquatic invasive plant species into the Great Lakes ecosystem (Lavole et al. 2003), it has also been responsible for the introduction of several forest pests (Yemshanov et al. 2011). The opening of the St. Lawrence Seaway also coincides with the initial reports of butternut canker in Wisconsin in 1967 (Renlund 1971). The introduction of isolates of *Oc-j* into different regions of North America may explain the geographic population subdivision identified using Hudson's *S*_nn_ test and verified in the AMOVA pairwise comparison of subdivided populations.

Given that butternut is not a dominant forest tree, benign or latent infections may have gone unnoticed or misdiagnosed for several years. For instance, the first reports of widespread butternut dieback were recorded in the 1920s (Graves 1919, 1923). However, the disease was attributed to *Melanconis juglandis*, even though the dieback symptoms could not be routinely replicated in greenhouse studies. It has since been demonstrated that these dieback symptoms were likely the result of an *Oc-j* infection and *M. juglandis* subsequently colonized the dead tissue (K. B. Broders, unpubl. data). In addition to causing the distinctive trunk cankers that lead to the mortality of butternut trees, *Oc-j* has been recovered from young stems, terminal buds, leaves, flowers, and seed pericarps and cotyledon on butternut, as well as branches and seed pericarps of black walnut and branches and flowers of heartnut (Broders and Boland 2010). Isolates of *Oc-j* have also been shown to vary in their virulence to butternut (Ostry and Moore 2008; Broders et al. 2011a). Given the above information, it is possible that isolates of *Oc-j* more commonly associated with anthracnose-like symptoms and dieback symptoms described by Graves (1919, 1923) were introduced into the northeastern United States and Canada around the turn of the century (Graves 1919). A more virulent strain may have been introduced into the upper Midwest via the Great Lakes and St. Lawrence Seaway causing the distinctive cankers that led to the initial diagnosis of butternut canker in the 1960s. This conclusion is based on the fact that isolates in genetic cluster 1, which is the dominant clonal lineage in the northeast, is more genetically diverse than isolates in genetic cluster 2 (Fig. 5), which are found at an equal frequency in the upper Midwest and Ontario, and are less common in the northeast (Fig. 7).

The genetic diversity and the structure of the host population is another factor that may have an important effect on the structure of the pathogen population. The distribution of genetic diversity of butternut in North America was attributed to range shift, which occurred after the last glacial retreat (∼1 bya) as southern populations began to recolonize areas previously covered by glaciers (Hoban et al. 2010). The populations of butternut in the northeastern United States are genetically distinct from those populations found in Minnesota and Wisconsin (Hoban et al. 2010). Another factor that may play a role in the ability of *Oc-j* populations to spread long distance is an expanded host range. The five isolates of *Oc-j* from black walnut and 1 isolate of *Oc-j* recovered from heartnut were all found to be members of all three genetic cluster (Table 1). Further genotyping and pathogenicity studies of isolates from black walnut and heartnut will determine the contribution of alternate hosts in the dispersal of *Oc-j*.

In summary, our findings that North American *Oc-j* populations cluster into three genetic groups provide new insight into the population genetic diversity and evolution of *Oc-j*, and support a model of multiple recent introduction or emergence events of *Oc-j* clonal lineages in North America. These findings also provide an example of how multiple introduction or emergence events are likely required for a pathogen to become resident in a new environment. In addition, once a new pathogen has established in a location on a novel host, there is still potential for a more virulent strain of the pathogen to emerge. Therefore, quarantine procedures may need to take into consideration not just the fact that the invasive pathogen has become established, but the likelihood of a more virulent strain being introduced and causing greater mortality than has already occurred. However, further genotyping studies are needed to provide a more complete picture of the host range, geographic range, and interactions among the clonal lineages. Additional pathogenicity experiments are also planned to evaluate the roles isolate virulence and host genotypes may play in the population structure of *Oc-j* and the dominance of specific clonal lineages in different regions of North America.

## References

[b1] Agapow PM, Burt A (2001). Indices of multilocus linkage disequilibrium. Mol. Ecol. Notes.

[b2] Anagnostakis SL (1987). Chestnut Blight: the classical problem of an introduced pathogen. Mycologia.

[b3] Arnaud-Haond S, Duarte CM, Alberto F, Serrao EA (2007). Standardizing methods to address clonality in population studies. Mol. Ecol.

[b4] Aylor DL, Price EW, Carbone I (2006). SNAP: combine and map modules for multilocus population genetic analysis. Bioinformatics.

[b5] Broders KD, Boland GJ (2010). Development of a molecular diagnostic assay for detection of the butternut canker pathogen *Sirococcus clavigignenti-juglandacearum*. Plant Dis.

[b6] Broders KD, Boland GJ (2011). Reclassification of the butternut canker fungus, *Sirococcus clavigignenti-juglandacearum*, into the genus *Ophiognomonia*. Fungal Biol.

[b7] Broders KD, Woeste K, SanMiguel PJ, Westerman RP, Boland GJ (2011a). Discovery of single nucleotide polymorphisms (SNPs) in the uncharacterized genome of the Ascomycete *Ophiognomonia clavigignenti-juglandacearum* from 454 sequence data. Mol. Ecol. Resour.

[b8] Broders K, Barbison L, Boland G (2011b). Population structure of *Ophiognomonia clavigignenti-juglandacearum* reveals multiple introductions of the butternut canker fungus into North America. Phytopathology.

[b9] Clement M, Posada D, Crandall KA (2000). TCS: a computer program to estimate gene genealogies. Mol. Ecol.

[b10] Couch BC, Fundal I, Lebrun MH, Tharreau D, Valent B (2005). Origins of host-specific populations of the blast pathogen *Magnoporthe oryzae* in crop domestication with subsequent expansion of pandemic clones on rice and weeds of rice. Genetics.

[b11] Davis CN, Myren DT, Czerwinski EJ (1992). First report of butternut canker in Ontario. Plant Dis.

[b12] Desprez-Loustau ML, Robin C, Buee M, Courtecuisse R, Garbaye J, Suffert F (2007). The fungal dimension of biological invasions. Trends Ecol. Evol.

[b101] Evanno G, Regnaut S, Goudet J (2005). Detecting the number of clusters of individuals using the software STRUCTURE: a simulation study. Mol. Ecol.

[b13] Excoffier L, Laval G, Schneider S (2005). Arlequin ver. 3.0: an integrated software package for population genetics data analysis. Evol. Bioinf. Online.

[b14] Furnier GR, Stolz AM, Mustaphi RM, Ostry ME (1999). Genetic evidence that butternut canker was recently introduced into North America. Can. J. Bot.

[b15] Graves AR (1919). Some diseases of trees in greater New York. Mycologia.

[b16] Graves AR (1923). The *Melanconis* disease of the butternut (*Juglans cinerea* L.). Phytopathology.

[b17] Halik S, Bergdahl DR (2002). Potential beetle vectors of *Sirococcus clavigignenti-juglandacearum* on butternut. Plant Dis.

[b18] Hall TA (1999). BioEdit: a user-friendly biological sequence alignment editor and analysis program for Window 95/98/NT. Nucleic Acids Symp. Ser.

[b19] Harrison KJ, Hurley JE, Ostry ME (1998). First report of butternut canker caused by *Sirococcus clavigignenti-juglandacearum* in New Brunswick, Canada. Plant Dis.

[b20] Hoban SM, Borkowski DS, Brosi SL, McCleary TS, Thompson LM, McLaughlin J (2010). Range-wide distribution of genetic diversity in the North Americn tree *Juglans cinerea*: a product of range shifts, not ecological marginaility or recent population decline. Mol. Ecol.

[b21] Hudson RR (2000). A new statistic for detecting genetic differntiation. Genetics.

[b22] Hudson RR, Kaplan NL (1985). Statistical properties of the number of recombination events in the history of a sample of DNA sequences. Genetics.

[b23] Hudson R, Boos D, Kaplan N (1992). A statistical test for detecting geographic subdivision. Mol. Biol. Evol.

[b24] Innes L, Rainville A (1996). Distribution and detection of *Sirococcus clavigignenti-juglandacearum* in Quebec. Phytoprotection.

[b25] Jombart T (2008). Adegenet: a R package for the multivariate analysis of genetic markers. Bioinfomatics.

[b26] Katovich SA, Ostry ME (1998). Insects associated with butternut and butternut canker in Minnesota and Wisconsin. The Great Lakes Entomol.

[b27] Kimura M (1983). The neutral theory of molecular evolution.

[b28] Lavole C, Jean M, Delisle F, Letourneau G (2003). Exotic plant species of the St. Lawrence River wetlands: a spatial and historical analysis. J. Biogeogr.

[b29] McKenna JR, Ostry ME, Woeste K (2011). Screening butternut and butternut hybrids for resistance to butternut canker.

[b30] Nair VMG, Kostichka CJ, Kuntz JE (1979). *Sirococcus clavigignenti-juglandacearum*: an undescribed species causing canker on butternut. Mycologia.

[b31] Neilson C, Cherry M, Boysen B, Hopkin A, McLaughlin J, Beardmore T (2003). COSEWIC status report on the butternut Juglans cinerea in Canada in COSEWIC assessment and status report on the butternut Juglans cinerea in Canada.

[b32] Orchard LP (1984).

[b33] Ostry ME (1997). *Sirococcus clavigignenti-juglandacearum* on heartnut (*Juglans ailantifolia* var. *cordiformis*. Plant Dis.

[b34] Ostry ME, Moore M (2007). Natural and experimental host range of *Sirococcus clavigignenti-juglandacearum*. Plant Dis.

[b35] Ostry ME, Moore M (2008). Response of butternut selections to inoculation with *Sirococcus clavigignenti-juglandacearum*. Plant Dis.

[b36] Ostry ME, Woeste K (2004).

[b37] Ostry ME, Katovich S, Anderson RL (1997). First report of *Sirococcus clavigignenti-juglandacearum* on black walnut. Plant Dis.

[b38] Parker IM, Gilbert GS (2004). The evolutionary ecology of novel plant-pathogen interactions. Annu. Rev. Ecol. Evol. Syst.

[b39] Pimentel D, Zuniga R, Morrison D (2005). Update on the environmental and economic costs associated with alien-invasive species in the United States. Ecol. Econ.

[b40] Price EW, Carbone I (2005). SNAP: workbench management tool for evolutionary population genetic analysis. Bioinformatics.

[b41] Pritchard JK, Stephens M, Donnelly P (2000). Inference of population structure using multilocus genotype data. Genetics.

[b42] Renlund DW (1971). Forest pest conditions in Wisconsin. Annual Report 1971.

[b43] Rozas J, Sánchez-DelBarrio JC, Messenguer X, Rozas R (2003). DnaSP, DNA polymorphism analyses by the coalescent and other methods. Bioinformatics.

[b44] Sakai AK, Allendorf FW, Holt JS, Lodge DM, Molofsky J, With KA (2001). The population biology of invasive species. Annu. Rev. Ecol. Syst.

[b45] Slatkin M (1994). Linkage disequilibrium in growing and stable populations. Genetics.

[b46] Sogonov MV, Castlebury LA, RA Y, Meija LC, White JF (2008). Leaf-inhabiting genera of the *Gnomoniaceae, Diapothathales*. Stud. Mycol.

[b47] Stewart JE, Halik S, Bergdahl DR (2004). Viability of *Sirococcus clavigignenti-juglandacearum* conidia on exoskeletons of three coleopteran species. Plant Dis.

[b48] Stukenbrock EH, McDonald BA (2008). The origins of plant pathogens in agro-ecosystems. Annu. Rev. Phytopathol.

[b49] Sun X, Guo LD, Hyde KD (2011). Community composition of endophytic fungi in *Acer truncatum* and their role in decomposition. Fungal Diversity.

[b50] Taylor JW, Jacobson DJ, Fisher MC (1999). The evolution of asexual fungi: reproduction, speciation, and classification. Annu. Rev. Phytopathol.

[b51] Thompson JD, Higgins DG, Gibson TJ (1994). Clustal W: improving the sensitivity of progressive multiple sequence alignment through sequence weighting, position-specific gap penalities and weight matrix choice. Nucleic Acids Res.

[b52] Yemshanov D, Koch FH, Ducey M, Koehler K (2011). Trade-associated pathways of alien forest insect entries in Canada. Biol. Invasions.

[b53] Zaffarano PL, McDonald BA, Linde CC (2008). Rapid speciation following recent host shifts in the plant pathogenic fungus Rhyncosporium. Evolution.

